# 4-{[(*E*)-(3,5-Dimethyl-1-phenyl-1*H*-pyrazol-4-yl)methyl­idene]amino}-1,5-dimethyl-2-phenyl-1*H*-pyrazol-3(2*H*)-one

**DOI:** 10.1107/S1600536810021173

**Published:** 2010-06-09

**Authors:** Hoong-Kun Fun, Madhukar Hemamalini, Abdullah M. Asiri, Salman A. Khan

**Affiliations:** a X-ray Crystallography Unit, School of Physics, Universiti Sains Malaysia, 11800 USM, Penang, Malaysia; b Department of Chemistry, Faculty of Science, King Abdu Aziz University, Jeddah, Saudi Arabia

## Abstract

The title Schiff base compound, C_23_H_23_N_5_O, was synthesized by the reaction of 4-amino­phenazone and 3,5-dimethyl-1-phenyl­pyrazole-4-carbaxaldehyde. The mol­ecule adopts an *E* configuration about the central C=N double bond. A weak intra­molecular C—H⋯O hydrogen bond generates an *S*(6) ring motif. The dihedral angle between the pyrazole rings is 24.72 (10)° and the dihedral angles between the pyrazole rings and the adjacent phenyl rings are 58.67 (10) and 46.58 (11)°. The crystal structure is stabilized by weak C—H⋯π inter­actions involving the pyrazolone and phenyl rings.

## Related literature

For background to and applications of heterocylic Schiff bases, see: Nawaz *et al.* (2009 ▶); Li *et al.* (1999 ▶); Urena *et al.* (2003 ▶); Geronikaki *et al.* (2003 ▶); Shanker *et al.* (2009 ▶); Pandeya *et al.* (1999 ▶); Sridhar *et al.* (2002 ▶); Nawrocka *et al.* (2004 ▶). For related structures, see: Eryigit & Kendi (1998 ▶); Manikandan *et al.* (2000 ▶). For details of hydrogen-bond motifs, see: Bernstein *et al.* (1995 ▶).
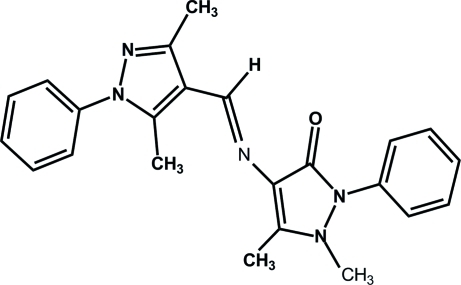


## Experimental

### 

#### Crystal data

C_23_H_23_N_5_O
*M* *_r_* = 385.46
Monoclinic, 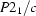

*a* = 15.2985 (2) Å
*b* = 7.6827 (1) Å
*c* = 19.6737 (3) Å
β = 116.905 (1)°
*V* = 2062.03 (5) Å^3^
*Z* = 4
Mo *K*α radiation
μ = 0.08 mm^−1^
*T* = 296 K
0.45 × 0.21 × 0.10 mm

#### Data collection

Bruker SMART APEXII CCD area-detector diffractometer
Absorption correction: multi-scan (*SADABS*; Bruker, 2009 ▶) *T*_min_ = 0.965, *T*_max_ = 0.992
23014 measured reflections
5993 independent reflections
2881 reflections with *I* > 2σ(*I*)
*R*_int_ = 0.048

#### Refinement

*R*[*F* ^2^ > 2σ(*F* ^2^)] = 0.059
*wR*(*F* ^2^) = 0.161
*S* = 1.03
5993 reflections
311 parameters
H atoms treated by a mixture of independent and constrained refinement
Δρ_max_ = 0.24 e Å^−3^
Δρ_min_ = −0.18 e Å^−3^

### 

Data collection: *APEX2* (Bruker, 2009 ▶); cell refinement: *SAINT* (Bruker, 2009 ▶); data reduction: *SAINT*; program(s) used to solve structure: *SHELXTL* (Sheldrick, 2008 ▶); program(s) used to refine structure: *SHELXTL*; molecular graphics: *SHELXTL*; software used to prepare material for publication: *SHELXTL* and *PLATON* (Spek, 2009 ▶).

## Supplementary Material

Crystal structure: contains datablocks global, I. DOI: 10.1107/S1600536810021173/lh5060sup1.cif

Structure factors: contains datablocks I. DOI: 10.1107/S1600536810021173/lh5060Isup2.hkl

Additional supplementary materials:  crystallographic information; 3D view; checkCIF report

## Figures and Tables

**Table 1:** Hydrogen-bond geometry (Å, °) *Cg*1 and *Cg*2 are the centroids of the N4/N5/C11–C13 and C1–C6 rings, respectively.

*D*—H⋯*A*	*D*—H	H⋯*A*	*D*⋯*A*	*D*—H⋯*A*
C10—H10*A*⋯O1	0.986 (18)	2.40 (2)	3.052 (3)	123.3 (14)
C19—H19*A*⋯*Cg*2^i^	0.990 (19)	2.656 (19)	3.452 (2)	137.4 (17)
C20—H20*C*⋯*Cg*1^ii^	0.96	2.85 (3)	3.720 (3)	149 (1)
C22—H22*B*⋯*Cg*2^iii^	0.96	2.82 (3)	3.585 (3)	135 (1)

Symmetry codes: (i) 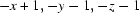; (ii) 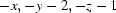; (iii) 
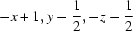
.

**Fig. 1.**
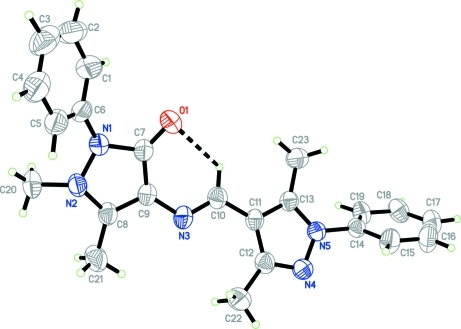
The asymmetric unit of the title compound, showing 50% probability displacement ellipsoids and the atom-numbering scheme. An intramolecular hydrogen bond is shown as dashed line.

**Fig. 2.**
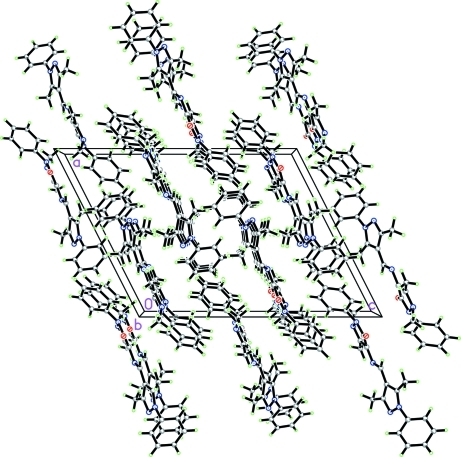
The crystal packing of the title compound showing showing the molecules stacked along b- axis.

## supplementary crystallographic information

### Comment

Heterocyclic Schiff bases have attracted continuing interest over the years because of their varied biological activities (Nawaz *et al.*, 2009). Recently they have found application in drug development for the treatment of allergies (Li *et al.*, 1999), hypertension (Urena *et al.*, 2003), inflammation (Geronikaki *et al.*, 20003), bacterial (Shanker *et al.*, 2009), HIV infections (Pandeya *et al.*, 1999) and hypnotics (Sridhar *et al.*, 2002). More recently they have also been used for the treatment of pain acting as fibrinogen receptor antagonists with antithrombotic activity (Nawrocka *et al.*, 2004). Due to wide application of pyrazoline-containing Schiff bases, we have synthesized a novel pyrazoline-containing Schiff base from 4-aminophenazone.

In the title compound (Fig. 1), the rings A (C14–C19), B (N4/N5/C11–C13), C (N1/N2/C7–C9) and D (C1–C6) are essentially planar.  The dihedral angle between the best planes of the rings are A/B = 58.67 (10)°, A/C = 83.07 (10)°, A/D = 79.53 (12)°, B/C = 24.72 (10)°, B/D = 44.68 (11)° and C/D = 46.58 (11)°. The molecule adopts a trans configuration about the central C10═N3 double bond. The C–N bond lengths of N1–C6 = 1.423 (2) Å; N2–C8 = 1.375 (2) Å; N3–C9 = 1.400 (2) Å; N5–C13 = 1.357 (2) Å; N1–C7 = 1.404 (2) Å; N2–C20 = 1.467 (2) Å; N4–C12 = 1.325 (2) Å and N5–C14 = 1.429 (2)  are normal for C–N single-bond distances. The distance between C10–N3 (1.287 (2) Å) is typical for a C═N double-bond distance. These bonds are comparable with those in *N*-(1*H*-benzoimidazol-2-ylmethyl)-*N*-(2,6-dichlorophenyl) amine (Eryigit & Kendi, 1998). The N1–N2 and N4–N5 (1.4082 (19) Å & 1.3702 (19) Å) single-bond lengths are comparable with those in 2,6-bis(3,5-dimethylpyrazol-1-ylmethyl) pyridine (Manikandan *et al.*, 2000). An weak intramolecular C10—H10A···O1 hydrogen bond interaction generates an *S*(6) ring motif (Bernstein *et al.*, 1995).

In the crystal structure (Fig. 2) there are no classical hydrogen bonds but stabilization is provided by weak C20—H20C···Cg1^i^, C19—H19A···Cg2^ii^ and C22—H22B···Cg2^iii^ interactions (see Table 1 for symmetry codes). Cg1 and Cg2 are the centroids of rings (N4/N5/C11–C13) and (C1–C6) rings respectively.

### Experimental

A mixture of 4-aminophenazone (0.50g, 0.0025 mol) and 3,5-dimethyl-1- phenylpyrazole-4-carbaxaldehyde (0.50 g, 0.0025 mol) in methanol (15 mL) was heated for 3 h to give a yellow precipitate.  It was then filtered and washed with methanol to gives the pure schiff bases ( I). Colourless crystals of (I) are recrystallized from methanol. Yield: 68%; m. p. 206°C. IR (KBr) ν_max_ cm^-1^: 2980 (C–H aromatic), 1642 (HC═N), 1607 (C═O), 1134 (C–N). ^1^H-NMR (600 MHz, CDCl_3_) d: 9.75 (CH═N, s), 7.49–7.29 (CH aromatic, m), 3.1(N–CH_3_, s), 2.84 (N-CH_3_, s), 2.15 (CH_3_, s), 1.70 (CH_3_, s).

### Refinement

Atoms H1A, H2A, H3A, H4A, H5A, H10A, H15A, H16A, H17A, H18A and H19A were located from a difference Fourier maps and refined freely. The methyl H atoms were positioned geometrically [C–H = 0.96 Å] and were refined using a riding model, with U_iso_(H) = 1.5U_eq_(C). A rotating group model was used for the methyl group.

### Crystal data

**Table:**

C_23_H_23_N_5_O	*F*(000) = 816
*M**_r_* = 385.46	*D*_x_ = 1.242 Mg m^−^^3^
Monoclinic, *P*2_1_/*c*	Mo *K*α radiation, λ = 0.71073 Å
Hall symbol: -P 2ybc	Cell parameters from 3425 reflections
*a* = 15.2985 (2) Å	θ = 2.8–22.2°
*b* = 7.6827 (1) Å	µ = 0.08 mm^−^^1^
*c* = 19.6737 (3) Å	*T* = 296 K
β = 116.905 (1)°	Block, colourless
*V* = 2062.03 (5) Å^3^	0.45 × 0.21 × 0.10 mm
*Z* = 4	

### Data collection

**Table:**

Bruker SMART APEXII CCD area-detector diffractometer	5993 independent reflections
Radiation source: fine-focus sealed tube	2881 reflections with *I* > 2σ(*I*)
graphite	*R*_int_ = 0.048
φ and ω scans	θ_max_ = 30.0°, θ_min_ = 1.5°
Absorption correction: multi-scan (*SADABS*; Bruker, 2009)	*h* = −20→21
*T*_min_ = 0.965, *T*_max_ = 0.992	*k* = −10→10
23014 measured reflections	*l* = −27→27

### Refinement

**Table:**

Refinement on *F*^2^	Secondary atom site location: difference Fourier map
Least-squares matrix: full	Hydrogen site location: inferred from neighbouring sites
*R*[*F*^2^ > 2σ(*F*^2^)] = 0.059	H atoms treated by a mixture of independent and constrained refinement
*wR*(*F*^2^) = 0.161	*w* = 1/[σ^2^(*F*_o_^2^) + (0.0683*P*)^2^ + 0.0093*P*] where *P* = (*F*_o_^2^ + 2*F*_c_^2^)/3
*S* = 1.03	(Δ/σ)_max_ < 0.001
5993 reflections	Δρ_max_ = 0.24 e Å^−^^3^
311 parameters	Δρ_min_ = −0.18 e Å^−^^3^
0 restraints	Extinction correction: *SHELXTL* (Sheldrick, 2008), Fc^*^=kFc[1+0.001xFc^2^λ^3^/sin(2θ)]^-1/4^
Primary atom site location: structure-invariant direct methods	Extinction coefficient: 0.0055 (13)

### Special details

**Table:**

Geometry. All esds (except the esd in the dihedral angle between two l.s. planes) are estimated using the full covariance matrix. The cell esds are taken into account individually in the estimation of esds in distances, angles and torsion angles; correlations between esds in cell parameters are only used when they are defined by crystal symmetry. An approximate (isotropic) treatment of cell esds is used for estimating esds involving l.s. planes.
Refinement. Refinement of F^2^ against ALL reflections. The weighted R-factor wR and goodness of fit S are based on F^2^, conventional R-factors R are based on F, with F set to zero for negative F^2^. The threshold expression of F^2^ > 2sigma(F^2^) is used only for calculating R-factors(gt) etc. and is not relevant to the choice of reflections for refinement. R-factors based on F^2^ are statistically about twice as large as those based on F, and R- factors based on ALL data will be even larger.

### Fractional atomic coordinates and isotropic or equivalent isotropic displacement parameters (Å^2^)

**Table:**

	*x*	*y*	*z*	*U*_iso_*/*U*_eq_	
O1	0.11416 (10)	0.47531 (17)	0.60512 (9)	0.0735 (5)	
N1	0.04112 (10)	0.21383 (18)	0.60925 (9)	0.0508 (4)	
N2	0.07320 (10)	0.04036 (17)	0.62683 (9)	0.0474 (4)	
N3	0.28754 (10)	0.22512 (19)	0.62644 (8)	0.0470 (4)	
N4	0.57171 (10)	0.41293 (19)	0.66310 (9)	0.0509 (4)	
N5	0.53154 (10)	0.55923 (17)	0.62050 (8)	0.0426 (4)	
C1	−0.09125 (16)	0.4099 (3)	0.58937 (15)	0.0687 (6)	
C2	−0.16074 (18)	0.4715 (4)	0.6111 (2)	0.0865 (9)	
C3	−0.17010 (19)	0.3990 (4)	0.6710 (2)	0.0855 (8)	
C4	−0.10854 (18)	0.2665 (3)	0.71182 (17)	0.0727 (7)	
C5	−0.03755 (15)	0.2068 (3)	0.69323 (13)	0.0568 (5)	
C6	−0.03009 (13)	0.2767 (2)	0.63100 (12)	0.0513 (5)	
C7	0.11729 (13)	0.3159 (2)	0.60932 (10)	0.0491 (5)	
C8	0.16367 (13)	0.0318 (2)	0.62798 (10)	0.0449 (4)	
C9	0.19323 (12)	0.1939 (2)	0.61851 (9)	0.0428 (4)	
C10	0.30947 (14)	0.3749 (2)	0.60912 (10)	0.0463 (4)	
C11	0.40715 (12)	0.4167 (2)	0.62077 (9)	0.0410 (4)	
C12	0.49667 (13)	0.3256 (2)	0.66248 (10)	0.0478 (5)	
C13	0.43302 (12)	0.5662 (2)	0.59479 (9)	0.0409 (4)	
C14	0.59465 (12)	0.6879 (2)	0.61384 (10)	0.0418 (4)	
C15	0.66808 (15)	0.7576 (3)	0.67935 (12)	0.0586 (5)	
C16	0.72986 (18)	0.8801 (3)	0.67306 (15)	0.0739 (7)	
C17	0.71962 (16)	0.9330 (3)	0.60346 (14)	0.0670 (6)	
C18	0.64705 (16)	0.8606 (3)	0.53863 (14)	0.0605 (6)	
C19	0.58412 (15)	0.7374 (2)	0.54338 (11)	0.0508 (5)	
C20	−0.00160 (14)	−0.0950 (2)	0.59196 (12)	0.0614 (5)	
H20A	−0.0387	−0.0723	0.5384	0.092*	
H20B	−0.0446	−0.0948	0.6154	0.092*	
H20C	0.0296	−0.2066	0.5992	0.092*	
C21	0.21737 (16)	−0.1350 (2)	0.64000 (15)	0.0748 (7)	
H21A	0.2829	−0.1125	0.6477	0.112*	
H21B	0.1844	−0.2079	0.5960	0.112*	
H21C	0.2196	−0.1927	0.6840	0.112*	
C22	0.51419 (15)	0.1564 (3)	0.70426 (13)	0.0727 (6)	
H22A	0.5833	0.1332	0.7302	0.109*	
H22B	0.4817	0.0645	0.6687	0.109*	
H22C	0.4889	0.1629	0.7408	0.109*	
C23	0.37292 (14)	0.7138 (3)	0.54809 (13)	0.0644 (6)	
H23A	0.4032	0.8218	0.5715	0.097*	
H23B	0.3084	0.7075	0.5448	0.097*	
H23C	0.3684	0.7073	0.4979	0.097*	
H1A	−0.0843 (13)	0.456 (2)	0.5486 (11)	0.055 (6)*	
H2A	−0.1957 (19)	0.562 (4)	0.5826 (15)	0.106 (9)*	
H3A	−0.2190 (17)	0.434 (3)	0.6873 (13)	0.088 (7)*	
H4A	−0.1115 (17)	0.210 (3)	0.7549 (14)	0.091 (8)*	
H5A	0.0095 (14)	0.115 (2)	0.7224 (11)	0.062 (6)*	
H10A	0.2610 (12)	0.469 (2)	0.5891 (10)	0.052 (5)*	
H15A	0.6715 (13)	0.724 (2)	0.7285 (11)	0.064 (6)*	
H16A	0.7790 (17)	0.924 (3)	0.7169 (14)	0.092 (8)*	
H17A	0.7596 (15)	1.023 (3)	0.5978 (12)	0.082 (7)*	
H18A	0.6394 (13)	0.892 (2)	0.4898 (12)	0.067 (6)*	
H19A	0.5326 (13)	0.682 (2)	0.4972 (11)	0.060 (5)*	

### Atomic displacement parameters (Å^2^)

**Table:**

	*U*^11^	*U*^22^	*U*^33^	*U*^12^	*U*^13^	*U*^23^
O1	0.0634 (9)	0.0410 (8)	0.1185 (13)	0.0000 (7)	0.0432 (9)	0.0135 (8)
N1	0.0454 (9)	0.0419 (8)	0.0686 (11)	−0.0020 (7)	0.0289 (8)	0.0045 (7)
N2	0.0494 (9)	0.0372 (8)	0.0616 (10)	−0.0071 (7)	0.0304 (8)	−0.0037 (7)
N3	0.0456 (9)	0.0494 (9)	0.0511 (9)	−0.0080 (7)	0.0263 (7)	−0.0023 (7)
N4	0.0458 (9)	0.0490 (9)	0.0560 (10)	−0.0008 (7)	0.0212 (7)	0.0111 (7)
N5	0.0415 (9)	0.0408 (8)	0.0467 (9)	−0.0013 (6)	0.0211 (7)	0.0051 (7)
C1	0.0535 (14)	0.0628 (14)	0.0775 (17)	0.0058 (11)	0.0188 (12)	0.0043 (12)
C2	0.0536 (15)	0.0703 (17)	0.113 (2)	0.0143 (13)	0.0180 (16)	−0.0166 (16)
C3	0.0572 (16)	0.093 (2)	0.109 (2)	−0.0042 (15)	0.0399 (16)	−0.0380 (18)
C4	0.0617 (15)	0.0759 (16)	0.0876 (18)	−0.0125 (13)	0.0400 (14)	−0.0276 (14)
C5	0.0496 (12)	0.0563 (12)	0.0661 (14)	−0.0056 (10)	0.0274 (11)	−0.0097 (11)
C6	0.0360 (10)	0.0458 (10)	0.0670 (13)	−0.0029 (8)	0.0189 (9)	−0.0075 (9)
C7	0.0427 (10)	0.0460 (10)	0.0557 (12)	−0.0055 (9)	0.0198 (9)	0.0049 (9)
C8	0.0464 (10)	0.0434 (10)	0.0523 (11)	−0.0062 (8)	0.0287 (9)	−0.0038 (8)
C9	0.0420 (10)	0.0455 (9)	0.0436 (10)	−0.0062 (8)	0.0216 (8)	−0.0023 (8)
C10	0.0465 (11)	0.0479 (10)	0.0465 (11)	−0.0042 (9)	0.0229 (9)	0.0013 (9)
C11	0.0428 (10)	0.0435 (9)	0.0420 (10)	−0.0061 (8)	0.0237 (8)	−0.0024 (8)
C12	0.0503 (11)	0.0479 (10)	0.0478 (11)	−0.0041 (9)	0.0243 (9)	0.0044 (8)
C13	0.0417 (10)	0.0425 (9)	0.0419 (10)	−0.0022 (7)	0.0218 (8)	−0.0005 (8)
C14	0.0415 (10)	0.0381 (9)	0.0508 (11)	−0.0025 (8)	0.0254 (9)	−0.0003 (8)
C15	0.0572 (12)	0.0649 (13)	0.0492 (13)	−0.0139 (10)	0.0200 (10)	0.0020 (10)
C16	0.0663 (15)	0.0719 (15)	0.0697 (17)	−0.0271 (12)	0.0187 (13)	−0.0036 (13)
C17	0.0657 (14)	0.0549 (12)	0.0818 (17)	−0.0181 (11)	0.0345 (13)	0.0019 (12)
C18	0.0769 (15)	0.0496 (11)	0.0662 (15)	−0.0079 (10)	0.0423 (13)	0.0047 (11)
C19	0.0615 (13)	0.0459 (10)	0.0501 (12)	−0.0115 (9)	0.0298 (10)	−0.0034 (9)
C20	0.0618 (13)	0.0536 (11)	0.0711 (14)	−0.0219 (10)	0.0322 (11)	−0.0095 (10)
C21	0.0783 (15)	0.0461 (12)	0.125 (2)	−0.0014 (11)	0.0680 (15)	−0.0048 (12)
C22	0.0644 (14)	0.0625 (13)	0.0870 (17)	0.0023 (11)	0.0306 (12)	0.0302 (12)
C23	0.0523 (12)	0.0575 (12)	0.0835 (15)	0.0062 (10)	0.0309 (11)	0.0151 (11)

### Geometric parameters (Å, °)

**Table:**

O1—C7	1.227 (2)	C11—C13	1.386 (2)
N1—C7	1.404 (2)	C11—C12	1.422 (2)
N1—N2	1.4082 (19)	C12—C22	1.496 (2)
N1—C6	1.423 (2)	C13—C23	1.487 (2)
N2—C8	1.375 (2)	C14—C19	1.375 (2)
N2—C20	1.467 (2)	C14—C15	1.378 (3)
N3—C10	1.287 (2)	C15—C16	1.379 (3)
N3—C9	1.400 (2)	C15—H15A	0.978 (19)
N4—C12	1.325 (2)	C16—C17	1.368 (3)
N4—N5	1.3702 (19)	C16—H16A	0.92 (2)
N5—C13	1.357 (2)	C17—C18	1.374 (3)
N5—C14	1.429 (2)	C17—H17A	0.96 (2)
C1—C6	1.378 (3)	C18—C19	1.383 (3)
C1—C2	1.396 (4)	C18—H18A	0.95 (2)
C1—H1A	0.927 (18)	C19—H19A	0.990 (19)
C2—C3	1.368 (4)	C20—H20A	0.9600
C2—H2A	0.90 (3)	C20—H20B	0.9600
C3—C4	1.372 (4)	C20—H20C	0.9600
C3—H3A	0.98 (2)	C21—H21A	0.9600
C4—C5	1.372 (3)	C21—H21B	0.9600
C4—H4A	0.97 (2)	C21—H21C	0.9600
C5—C6	1.388 (3)	C22—H22A	0.9600
C5—H5A	0.984 (19)	C22—H22B	0.9600
C7—C9	1.439 (2)	C22—H22C	0.9600
C8—C9	1.366 (2)	C23—H23A	0.9600
C8—C21	1.483 (2)	C23—H23B	0.9600
C10—C11	1.442 (2)	C23—H23C	0.9600
C10—H10A	0.982 (17)		
			
C7—N1—N2	109.36 (13)	C11—C12—C22	129.15 (16)
C7—N1—C6	123.92 (15)	N5—C13—C11	106.51 (14)
N2—N1—C6	118.45 (14)	N5—C13—C23	122.19 (15)
C8—N2—N1	106.59 (13)	C11—C13—C23	131.30 (16)
C8—N2—C20	122.69 (15)	C19—C14—C15	120.63 (17)
N1—N2—C20	116.40 (14)	C19—C14—N5	120.55 (16)
C10—N3—C9	120.18 (15)	C15—C14—N5	118.79 (16)
C12—N4—N5	105.24 (14)	C14—C15—C16	118.9 (2)
C13—N5—N4	112.08 (13)	C14—C15—H15A	118.6 (11)
C13—N5—C14	128.42 (14)	C16—C15—H15A	122.4 (11)
N4—N5—C14	119.27 (13)	C17—C16—C15	121.4 (2)
C6—C1—C2	118.4 (3)	C17—C16—H16A	120.3 (15)
C6—C1—H1A	119.1 (12)	C15—C16—H16A	118.3 (15)
C2—C1—H1A	122.5 (12)	C16—C17—C18	119.2 (2)
C3—C2—C1	121.1 (3)	C16—C17—H17A	122.6 (13)
C3—C2—H2A	125.9 (17)	C18—C17—H17A	118.2 (13)
C1—C2—H2A	113.0 (18)	C17—C18—C19	120.6 (2)
C2—C3—C4	119.6 (3)	C17—C18—H18A	121.0 (12)
C2—C3—H3A	124.3 (14)	C19—C18—H18A	118.4 (12)
C4—C3—H3A	116.1 (14)	C14—C19—C18	119.34 (19)
C5—C4—C3	120.6 (3)	C14—C19—H19A	119.2 (10)
C5—C4—H4A	115.9 (14)	C18—C19—H19A	121.5 (10)
C3—C4—H4A	123.5 (14)	N2—C20—H20A	109.5
C4—C5—C6	119.8 (2)	N2—C20—H20B	109.5
C4—C5—H5A	122.7 (11)	H20A—C20—H20B	109.5
C6—C5—H5A	117.5 (11)	N2—C20—H20C	109.5
C1—C6—C5	120.4 (2)	H20A—C20—H20C	109.5
C1—C6—N1	118.7 (2)	H20B—C20—H20C	109.5
C5—C6—N1	120.90 (17)	C8—C21—H21A	109.5
O1—C7—N1	123.40 (17)	C8—C21—H21B	109.5
O1—C7—C9	131.56 (17)	H21A—C21—H21B	109.5
N1—C7—C9	104.99 (14)	C8—C21—H21C	109.5
C9—C8—N2	110.30 (15)	H21A—C21—H21C	109.5
C9—C8—C21	128.03 (16)	H21B—C21—H21C	109.5
N2—C8—C21	121.65 (15)	C12—C22—H22A	109.5
C8—C9—N3	121.95 (15)	C12—C22—H22B	109.5
C8—C9—C7	108.22 (15)	H22A—C22—H22B	109.5
N3—C9—C7	129.41 (15)	C12—C22—H22C	109.5
N3—C10—C11	122.19 (18)	H22A—C22—H22C	109.5
N3—C10—H10A	121.7 (10)	H22B—C22—H22C	109.5
C11—C10—H10A	116.1 (10)	C13—C23—H23A	109.5
C13—C11—C12	105.06 (14)	C13—C23—H23B	109.5
C13—C11—C10	125.06 (16)	H23A—C23—H23B	109.5
C12—C11—C10	129.81 (16)	C13—C23—H23C	109.5
N4—C12—C11	111.10 (15)	H23A—C23—H23C	109.5
N4—C12—C22	119.73 (16)	H23B—C23—H23C	109.5
			
C7—N1—N2—C8	−7.52 (18)	N1—C7—C9—C8	−3.59 (19)
C6—N1—N2—C8	−157.20 (15)	O1—C7—C9—N3	1.0 (3)
C7—N1—N2—C20	−148.51 (16)	N1—C7—C9—N3	−176.21 (16)
C6—N1—N2—C20	61.8 (2)	C9—N3—C10—C11	176.07 (15)
C12—N4—N5—C13	1.35 (18)	N3—C10—C11—C13	171.42 (16)
C12—N4—N5—C14	176.16 (14)	N3—C10—C11—C12	−12.0 (3)
C6—C1—C2—C3	−1.7 (4)	N5—N4—C12—C11	−1.32 (19)
C1—C2—C3—C4	1.7 (4)	N5—N4—C12—C22	−179.90 (16)
C2—C3—C4—C5	0.3 (4)	C13—C11—C12—N4	0.84 (19)
C3—C4—C5—C6	−2.3 (3)	C10—C11—C12—N4	−176.23 (17)
C2—C1—C6—C5	−0.3 (3)	C13—C11—C12—C22	179.26 (19)
C2—C1—C6—N1	−179.76 (19)	C10—C11—C12—C22	2.2 (3)
C4—C5—C6—C1	2.3 (3)	N4—N5—C13—C11	−0.84 (18)
C4—C5—C6—N1	−178.27 (17)	C14—N5—C13—C11	−175.06 (15)
C7—N1—C6—C1	62.0 (2)	N4—N5—C13—C23	179.18 (16)
N2—N1—C6—C1	−153.01 (17)	C14—N5—C13—C23	5.0 (3)
C7—N1—C6—C5	−117.4 (2)	C12—C11—C13—N5	0.01 (17)
N2—N1—C6—C5	27.5 (2)	C10—C11—C13—N5	177.27 (15)
N2—N1—C7—O1	−170.72 (18)	C12—C11—C13—C23	179.99 (19)
C6—N1—C7—O1	−23.1 (3)	C10—C11—C13—C23	−2.8 (3)
N2—N1—C7—C9	6.80 (18)	C13—N5—C14—C19	−63.0 (2)
C6—N1—C7—C9	154.47 (17)	N4—N5—C14—C19	123.15 (18)
N1—N2—C8—C9	5.20 (19)	C13—N5—C14—C15	118.9 (2)
C20—N2—C8—C9	143.14 (17)	N4—N5—C14—C15	−55.0 (2)
N1—N2—C8—C21	−176.14 (18)	C19—C14—C15—C16	1.1 (3)
C20—N2—C8—C21	−38.2 (3)	N5—C14—C15—C16	179.18 (18)
N2—C8—C9—N3	172.28 (14)	C14—C15—C16—C17	−0.1 (3)
C21—C8—C9—N3	−6.3 (3)	C15—C16—C17—C18	−0.9 (4)
N2—C8—C9—C7	−1.0 (2)	C16—C17—C18—C19	0.9 (3)
C21—C8—C9—C7	−179.6 (2)	C15—C14—C19—C18	−1.1 (3)
C10—N3—C9—C8	172.48 (17)	N5—C14—C19—C18	−179.16 (16)
C10—N3—C9—C7	−15.8 (3)	C17—C18—C19—C14	0.1 (3)
O1—C7—C9—C8	173.6 (2)		

### Hydrogen-bond geometry (Å, °)

**Table:**

Cg1 and Cg2 are the centroids of the N4/N5/C11–C13 and C1–C6 rings, respectively.

**Table:**

*D*—H···*A*	*D*—H	H···*A*	*D*···*A*	*D*—H···*A*
C10—H10A···O1	0.986 (18)	2.40 (2)	3.052 (3)	123.3 (14)
C19—H19A···Cg2^i^	0.990 (19)	2.656 (19)	3.452 (2)	137.4 (17)
C20—H20C···Cg1^ii^	0.96	2.85 (3)	3.720 (3)	149 (1)
C22—H22B···Cg2^iii^	0.96	2.82 (3)	3.585 (3)	135 (1)

Symmetry codes: (i) −*x*+1, −*y*−1, −*z*−1; (ii) −*x*, −*y*−2, −*z*−1; (iii) −*x*+1, *y*−1/2, −*z*−1/2.
